# Natural phytochemical-based strategies for antibiofilm applications

**DOI:** 10.1186/s13020-025-01147-5

**Published:** 2025-07-01

**Authors:** Kangyu Zhou, Mengyao Shi, Ruyi Chen, Yang Zhang, Yunjie Sheng, Chaoying Tong, Gang Cao, Dan Shou

**Affiliations:** 1 School of Pharmaceutical Sciences, Zhejiang Chinese Medical University, Hangzhou, 310053 Zhejiang People’s Republic of China; 2 Institute of Orthopedics and Traumatology, The First Affiliated Hospital of Zhejiang Chinese Medical University, Hangzhou, 310053 Zhejiang People’s Republic of China

**Keywords:** Biofilms, Phytochemicals, Natural products, Nanomaterials, Antibiofilm activity

## Abstract

**Background:**

Biofilms contribute to the persistence of infectious diseases, complicate the treatment of chronic infections and pose a significant global health threat. However, the effectiveness of antibacterial therapies is often limited by poor penetration of antibiotics, as well as the horizontal transfer of antibiotic resistance genes among bacteria. Phytochemicals remain a promising source for developing novel antibiofilm agents.

**Methods:**

A systematic search of literatures was conducted using PubMed, Web of Science, Google scholar, and CNKI, with keywords related to “phytochemicals”, “natural products”, “natural compounds”, “alkaloids”, “polyphenols”, “terpenoids”, “quinones”, “nanomaterials”, “biofilms”, “biofilm formation”, “biofilm inhibition”, and “structure–activity relationship” focusing on studies published from 2014 to 2025.

**Results:**

A total of 38 most extensively studied natural phytochemicals, including alkaloids, flavonoids (i.e., flavonols, flavanols, and chalcones), quinones, non-flavonoid polyphenols, terpenes and others, were systematically screened based on relevant articles from the past decade. Phytochemicals mainly work by targeting quorum sensing systems, reducing virulence factor production, preventing the initial adhesion and targeting the extracellular polymeric substances of biofilms. Well-designed phytochemical-based nanomaterials can enhance permeability, drug loading efficiency, target drug delivery and sustained drug release of phytochemicals, thereby increasing their antibiofilm efficacy.

**Conclusion:**

Phytochemicals represent a promising therapeutic source for the elimination of bacterial biofilms and associated infections both in the form of molecules or nanomaterials. By synthesizing current progress and identifying future directions, phytochemical-based strategies may inspire innovative solutions and promote translational efforts in combating biofilm-associated challenges in clinical and environmental contexts.

**Graphical abstract:**

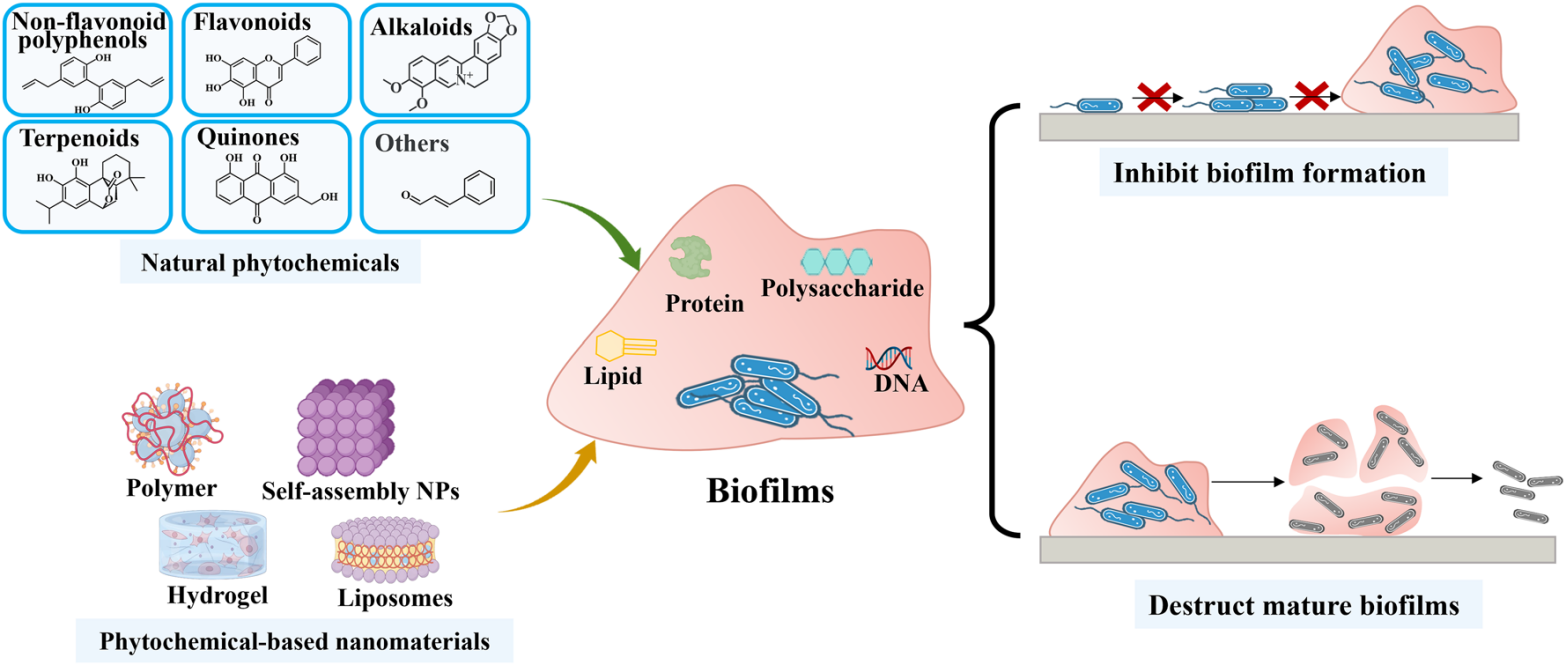

## Introduction

Chronic microbial infections represent a significant and growing challenge to global healthcare systems. These infections are often characterized by their refractory and persistent nature, as exemplified by intractable diseases such as osteomyelitis and cellulitis. A crucial contributor to this therapeutic challenge is biofilms. The biofilms are structured microorganism communities that adhere to surfaces or are non-surface-attached, which exhibit heightened resistance to antibiotics and help bacteria evade the host immune response [1]. Indeed, bacteria embedded in biofilms can display up to a 1000-fold reduction in antibiotic susceptibility compared to their planktonic counterparts, rendering conventional treatments largely ineffective [2]. This remarkable resistance significantly contributes to the recalcitrance of biofilm-associated infections and complicates eradication efforts using standard antimicrobial therapies. For the reason of lacking effective agents for eradicating biofilm-associated infections, researchers are exploring alternative therapeutic strategies, with natural products emerging as a focus due to their diverse pharmacological activities including antibiofilm, antibacterial, anti-inflammatory, and immunomodulatory properties.

It is reported that plant extracts are effective for eliminating bacterial biofilms and their associated infections [3]. However, the content and composition of active ingredients are affected by the environment, origin, weather, extraction technology, etc. [4]. Besides, the unclear composition complicates the development of targeted therapy, particularly in controlling drug dosage, efficacy, and side effects. Furthermore, certain toxic compounds in plants exacts could present health risks, requiring more rigorous toxicological evaluations and usage guidelines [5]. Phytochemicals are specific compounds extracted from plants, with clear molecular structures and distinct biological activities. Their pharmacological mechanisms are easier to study, thus allowing for more precise therapy and new drug design. Historically, natural chemicals have served as a cornerstone for clinical drug discovery, with 32% of newly approved small-molecule drugs (between 1981 and 2019) were either directly derived from or structurally inspired by natural phytochemicals or their derivatives [6]. Up to now, various phytochemicals have been proved to have significant effects in inhibiting and disrupting biofilms. For example, quercetin, curcumin (Cur), and berberine (BBR) show remarkable efficacy in inhibiting biofilm formation and reducing bacterial activity within biofilms. Compared to conventional antibiotics, phytochemicals can offer distinct advantages due to their multitargeted antibacterial mechanisms, including direct bactericidal or bacteriostatic effects, modulation of virulence factors and interference with quorum sensing (QS) systems. This broad spectrum of activity significantly reduces the likelihood of resistance development, which is a major concern in prolonged antibiotic therapy. Moreover, small-molecule phytochemicals can effectively overcome the antibiotic tolerance conferred by the biofilm phenotype [7]. Consequently, phytochemicals hold great promise not only as standalone antibiofilm agents but also as adjuvants in combination therapies that enhance the efficacy of existing antibiotics.

Despite the therapeutic potential, clinical translation of phytochemicals is often hindered by poor solubility and bioavailability [8]. Consequently, the integration of advanced nanotechnology to enhance the therapeutic efficacy of phytochemical-based treatments has emerged as an essential direction in biomedical research [9]. Nanomaterials possess adjustable particle size, shape, and surface characteristics [10], which are beneficial for rational design of phytochemicals for target-biofilm delivery, minimizing side effects, and responsive release [11]. By combining the biocompatibility and bioactivity of phytochemicals with the design flexibility of nanotechnologies, natural phytochemical-based nanomaterials can synergistically improve tissue penetration and enable targeted release. As a result, these nanocarriers achieve higher drug concentrations at infection sites and exhibit enhanced antibiofilm activity.

Numerous reviews on biofilms have covered topics such as the clinical applications of plant-derived antibiofilm strategies [3] and the use of nanotechnologies to penetrate bacterial biofilm matrix barriers [12]. However, few studies have specifically focused on the natural phytochemical-based antibiofilm strategies.

For the purpose of providing new insights into phytochemicals and guiding the discovery of biofilm-related drugs, This review focuses on alkaloids, flavonoids, terpenoids, quinones, and non-flavonoid polyphenols, with well-documented antimicrobial and antibiofilm properties. It summarizes the characteristics of biofilms over their development cycle, highlights the latest advancements in the antibiofilm applications of phytochemicals, including bioactive compounds and phytochemical-based nanomaterials, overviews their mechanisms of action and clinical potential for developing novel nanomedicines in depth, and finally offers the prospects and challenges of natural phytochemicals applying in the field of antibiofilm.

### Methodology

To collect relevant data, an extensive literature search was conducted across multiple electronic databases, including PubMed, Web of Science, China National Knowledge Infrastructure (CNKI), covering publications up until 2025. The search strategy was designed to identify studies related to the antibiofilm effects of plant-derived compounds.

Using the PubMed database as an example, the following combination of MeSH terms and free-text keywords was used: ("Phytochemicals" [Mesh] OR "Natural Products" [Mesh] OR "Natural Compounds" OR "Flavonoids" [Mesh] OR "Alkaloids" [Mesh] OR "Polyphenols" [Mesh] OR "Terpenes" [Mesh] OR "Quinones" [Mesh]) AND ("Biofilm" [Mesh] OR "Biofilm Formation" OR "Biofilm Inhibition"). Only publications written in English were considered.

### Eligibility criteria

(i) The study investigated phytochemicals for their antibiofilm activity. (ii) The research involved quantitative assessment of antibiofilm efficacy using parameters such as the minimum biofilm inhibitory concentration (MBIC), minimum biofilm eradication concentration (MBEC), minimum inhibitory concentration (MIC), and minimum bactericidal concentration (MBC). (iii) The study included relevant mechanistic investigations, such as analysis of quorum sensing pathways, virulence gene expression, EPS disruption, or biofilm architecture modulation. (iv) Peer-reviewed original research articles published between 2014 and 2025.

### Exclusion criteria

(i) Editorials, reviews, clinical studies, theoretical research, conference abstracts, book chapters, letters, and patents. (ii) Studies that did not meet the above criteria, particularly those lacking MBIC/MBEC or MIC/MBC data or mechanistic analysis. (iii) Research based on crude extracts, multi-component traditional herbal formulations, or complex plant mixtures where the active constituents were not clearly defined or separated. (iv) Papers solely focusing on planktonic antimicrobial activity without evaluating effects on biofilm formation or disruption.

## Overview of biofilms

### Biofilm formation

**Fig. 1:**
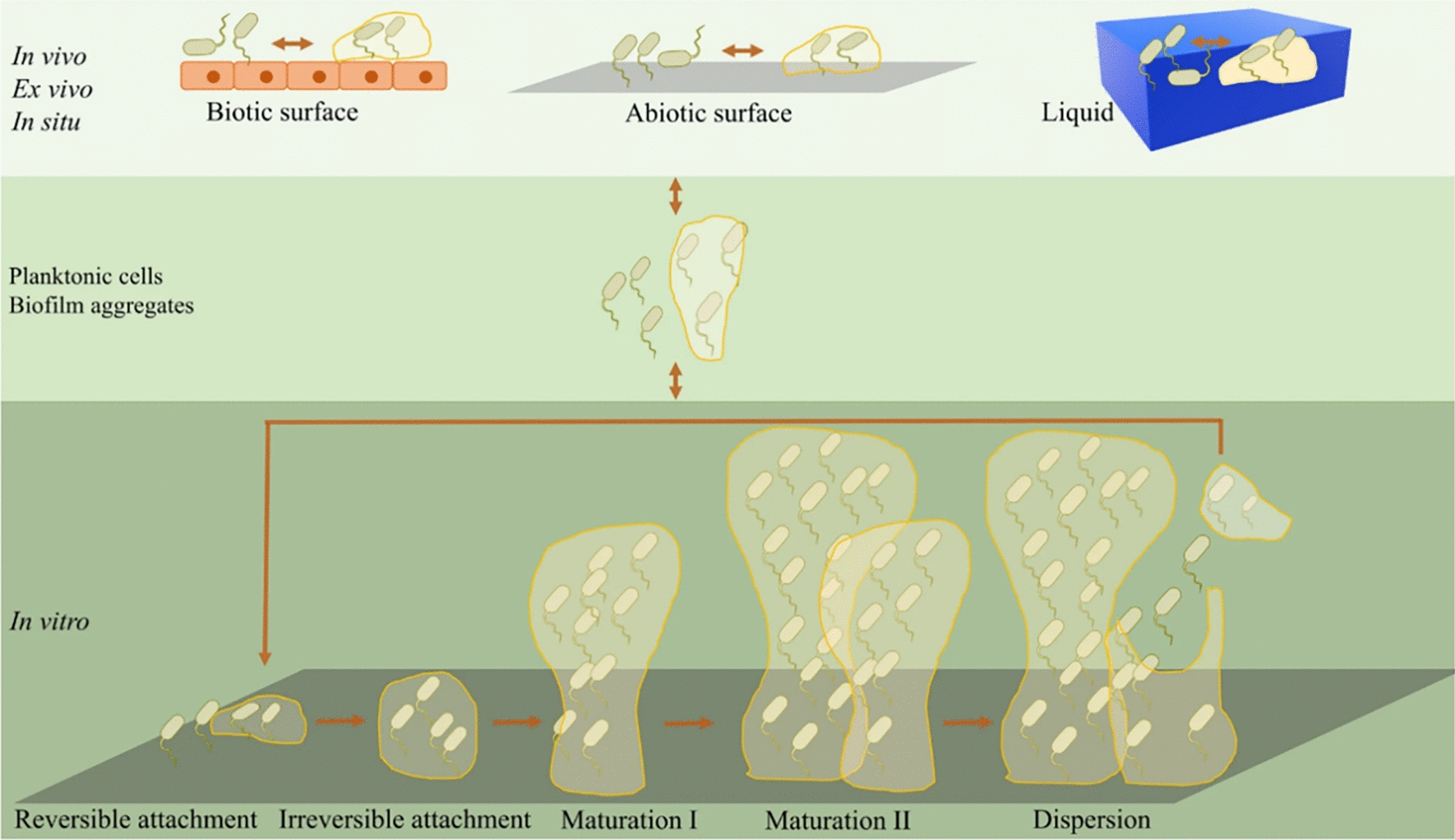
Expanded biofilm formation model [26]. Copyright 2023, Springer Nature. This work is licensed under CC BY 4.0. The commonly accepted in vitro biofilm formation model developed by *P. aeruginosa* (Five major stages consisting of reversible attachment, irreversible attachment, maturation I, maturation II, and dispersion)

Biofilms are structured aggregates of microorganisms that adhere to biotic or abiotic surfaces through the secretion of extracellular polymeric substances (EPS) [13]. Biofilm formation is a cyclic process influenced by environmental factors, which modulate microbial gene expression and drive phenotypic transitions during different developmental stages. The composition of the EPS matrix varies considerably between bacterial and fungal biofilms. In bacterial biofilms, the EPS typically contains proteins, polysaccharides, extracellular DNA (eDNA), and lipids, which interact to form a cohesive three-dimensional network that facilitates cell adhesion, biofilm architecture, and resistance to environmental stress [14]. In contrast, fungal biofilms, such as those formed by Candida albicans, produce a matrix rich in β-1,3-glucan, mannan, lipids, proteins, and DNA [15]. These components not only contribute to the structural integrity of the fungal biofilm but also bind to and neutralize antifungal agents, thus enhancing drug resistance. Furthermore, bacteria generally exhibit uniform cell shapes, such as cocci or rods, while fungi display pronounced morphological polymorphism, including yeast, pseudohyphal, and hyphal forms [16]. The coexistence of these diverse morphologies within biofilm adds to the architectural complexity and contributes to enhanced resistance against antifungal agents and host immune defenses [17]. Therefore, understanding the compositional and structural differences between bacterial and fungal EPS matrices is critical for designing more effective, targeted strategies to combat biofilm-associated infections. The most widely accepted model for bacterial biofilm formation is based on in vitro studies of *Pseudomonas aeruginosa*, and it consists of five major stages (Fig. 1) [18, 19]:

(i) Reversible attachment. Planktonic bacteria initially adhere to the substratum at the cell pole or via the flagellum. This first surface contact is based on van der Waals forces (electrostatic, hydrophobic interactions, etc.), which is unstable and reversible, with bacteria frequently returning to the liquid phase [19].
(ii) Irreversible attachment. Bacteria are firmly adhered to the substratum with newly formed cell clusters. They cease to move and cement themselves to the surface or to each other more firmly through initiating the matrix production [18]. The transition to this stage is marked by the downregulation of flagellar gene expression and the upregulation of genes responsible for the biosynthesis of the Psl matrix polymer [20]. Additionally, this phase involves the activation of genes(i.e., *β*-lactamase, phenazine, SagS and BrlR) associated with antibiotic resistance [21–23].
(iii) Maturation I. The first biofilm maturation stage is characterized by the appearance of cell clusters that are several-cells thick and are embedded in the biofilm matrix.
(iv) Maturation II. Bacteria grow into a more complex multicellular mature form, which is characterized by the presence of differentiated, mushroom-like or pillar-like structures or microcolonies interspersed with fluid-filled channels [24]. This micro-structure is determined by the interplay between intrinsic bacterial regulation and environmental conditions. Factors such as cellular crowding, chemical gradients, and nutrient competition drive the stratification of the biofilm and facilitate the emergence of distinct microbial subpopulations [25].
(v) Dispersion. Dispersion is widely recognized as the terminal phase of biofilm development. In this stage, bacteria can depart from the biofilm and return to the planktonic mode of growth, which facilitates a new cycle of biofilm development at new sites of colonization [19].

The biofilm formation is mainly controlled in genetic-regulation way through three aspects [27]: (i) QS. Bacteria regulate gene expression via the QS system, which detects bacterial population density and produces signaling molecules [28]. When these molecules reach a critical concentration, QS mechanism is triggered, coordinating gene expression and enabling the bacteria to cooperate and form the biofilm. (ii) Cyclic dinucleotides. Cyclic dimeric guanosine monophosphate (c-di-GMP) as a representative cyclic dinucleotide, can modulate the expression of genes involved in biofilm formation [29]. Elevated level of c-di-GMP typically promote the synthesis and secretion of EPS, strengthening the structure and functionality of biofilms [30]. (iii) Small non-coding RNAs. Small non-coding RNAs, another key factor in biofilm formation, affecting gene expression and metabolic pathways within bacteria [31] to influence various physiological states of matrix shields (such as stationary, slow-growing, or low-metabolic states), which further contribute to their resistance to stresses from immune cells, environmental, and conventional antibiotics [32].

### Biofilm-related clinical diseases and therapeutic challenges

There are two types of diseases closely related to biofilms: clinical and medical device-associated disorders, and non-device-associated chronic disorders. Implantable medical devices (i.e., pacemakers and artificial joints) have become an indispensable component of modern medicine for improving the the quality of life for patients. However, the incidence of medical device-associated infections has increased in recent years. Examples include urinary catheter-related urinary system infections in urology [33], biomaterial artificial valve implantation in cardiac surgery [34], orthopaedic artificial joint implantation [35], middle ear trachea replacement implantation implants in otorhinolaryngology [36], the application of medical implants in orthopaedic surgery [37] and other implant infections. Non-device-associated chronic diseases are characterised by the formation of biofilm on the surface or inside an injured tissue cavity [38]. Common chronic infections include chronic osteomyelitis, pulmonary cystic fibrosis, chronic sinusitis, periodontitis, and dental plaques [39].

**Fig. 2:**
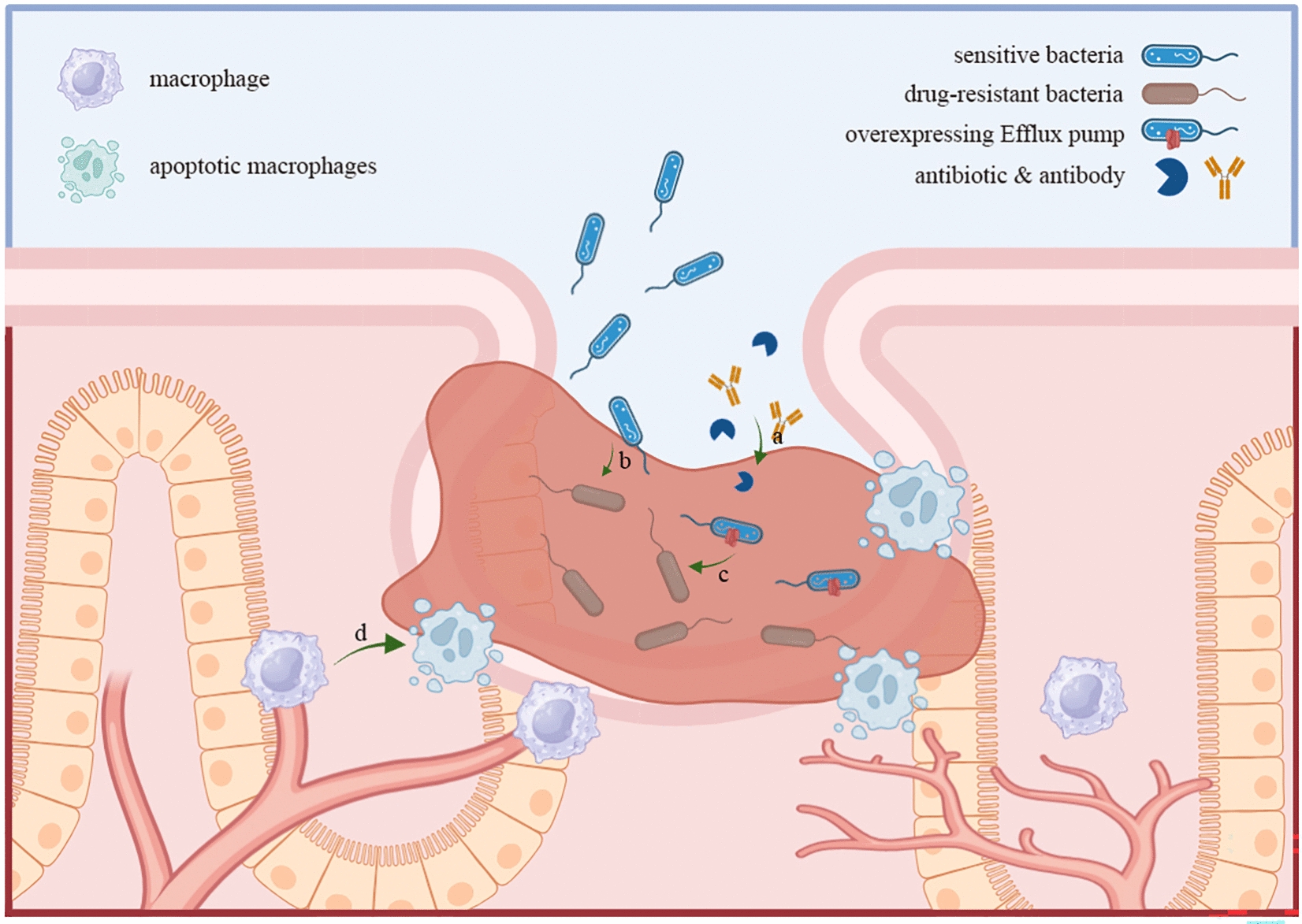
Schematic diagram of bacterial biofilm resistance mechanism. **a** Reducing antibiotic permeability due to the biofilm matrix barrier. **b** Facilitating lateral transfer of drug resistance genes under nutrient-deficient and low metabolic conditions in the biofilm microenvironment. **c** Producing bacterial efflux pumps to expel antibiotics. **d** Weakening and interfering with the host immune system by destroying immune cells and disrupting immune cell receptors

Long-term, chronic bacterial infections and antibiotic resistance are major challenges associated with the biofilm-related clinical diseases [40]. Bacteria within biofilms interact to produce toxins and activate the host immune system to trigger the inflammatory response, leading to the persistence of chronic inflammation or infection [41]. Bacteria may also spread to surrounding tissues through biofilm ducts, exacerbating the infection [42]. Besides, the biofilm matrix, with its strong adherence to surfaces, exhibits significant barrier and hydrophobic property. These attributes hinder the effective penetration of antibiotics and nutrients, consequently reduce the metabolic rate of bacteria and enhance their protection [43]. Moreover, long-term antibiotic treatment may eventually lead to liver damage and systemic failure in patients, thereby endangering their lives [44]. Therefore, overcoming the obstacles in biofilm therapy is one of the main challenges in the treatment of biofilm-related diseases. The mechanism of biofilm resistance is illustrated in Fig. 2.

## Natural phytochemicals for biofilm-infection treatment

**Fig. 3:**
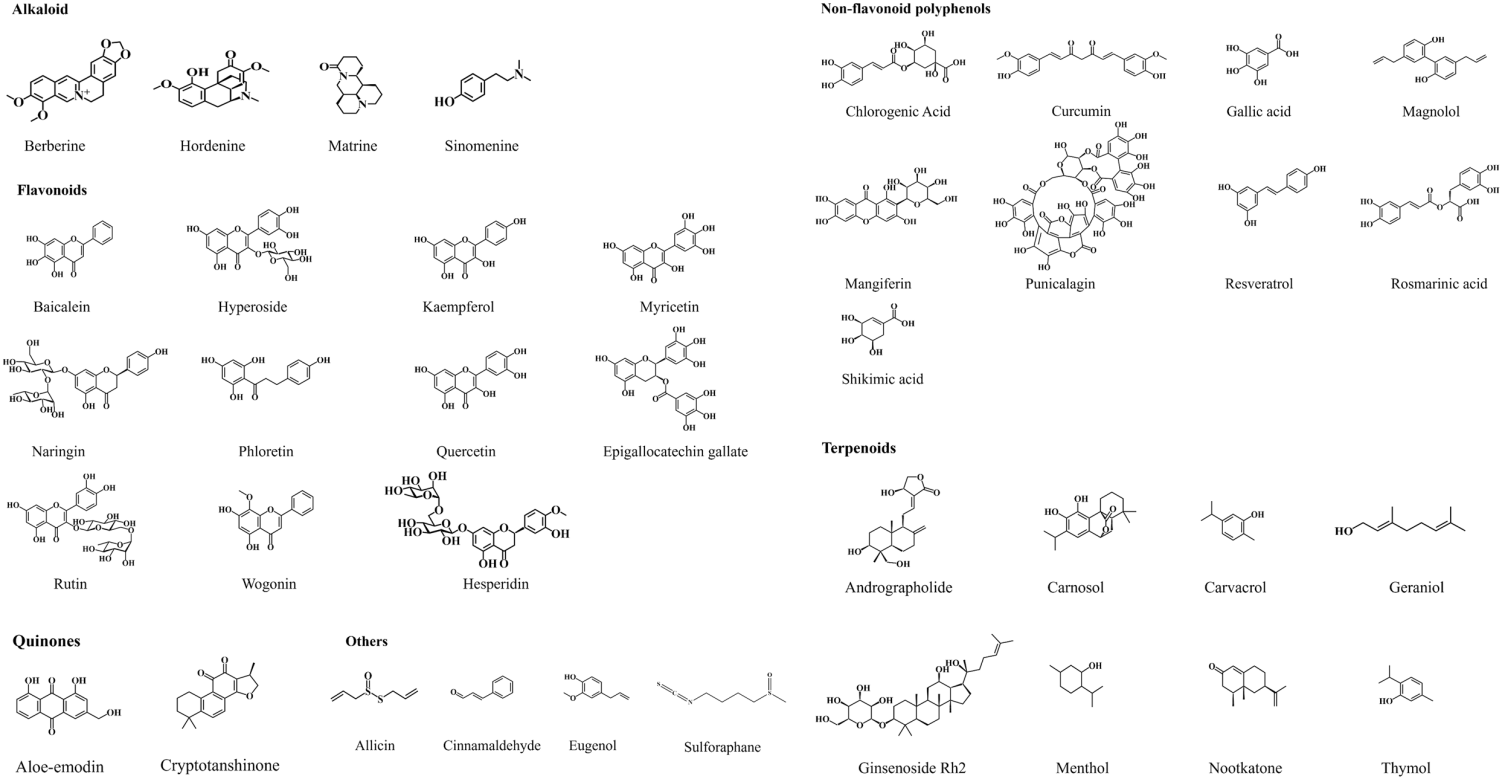
Types of natural phytochemicals with antibiofilm potential. Natural phytochemicals are broadly classified into several structural groups, including alkaloids, flavonoids, terpenoids, quinones, non-flavonoid polyphenols and others. Many of these compounds exhibit antimicrobial and antibiofilm properties, making them promising candidates for novel therapeutic strategies

**Fig. 4:**
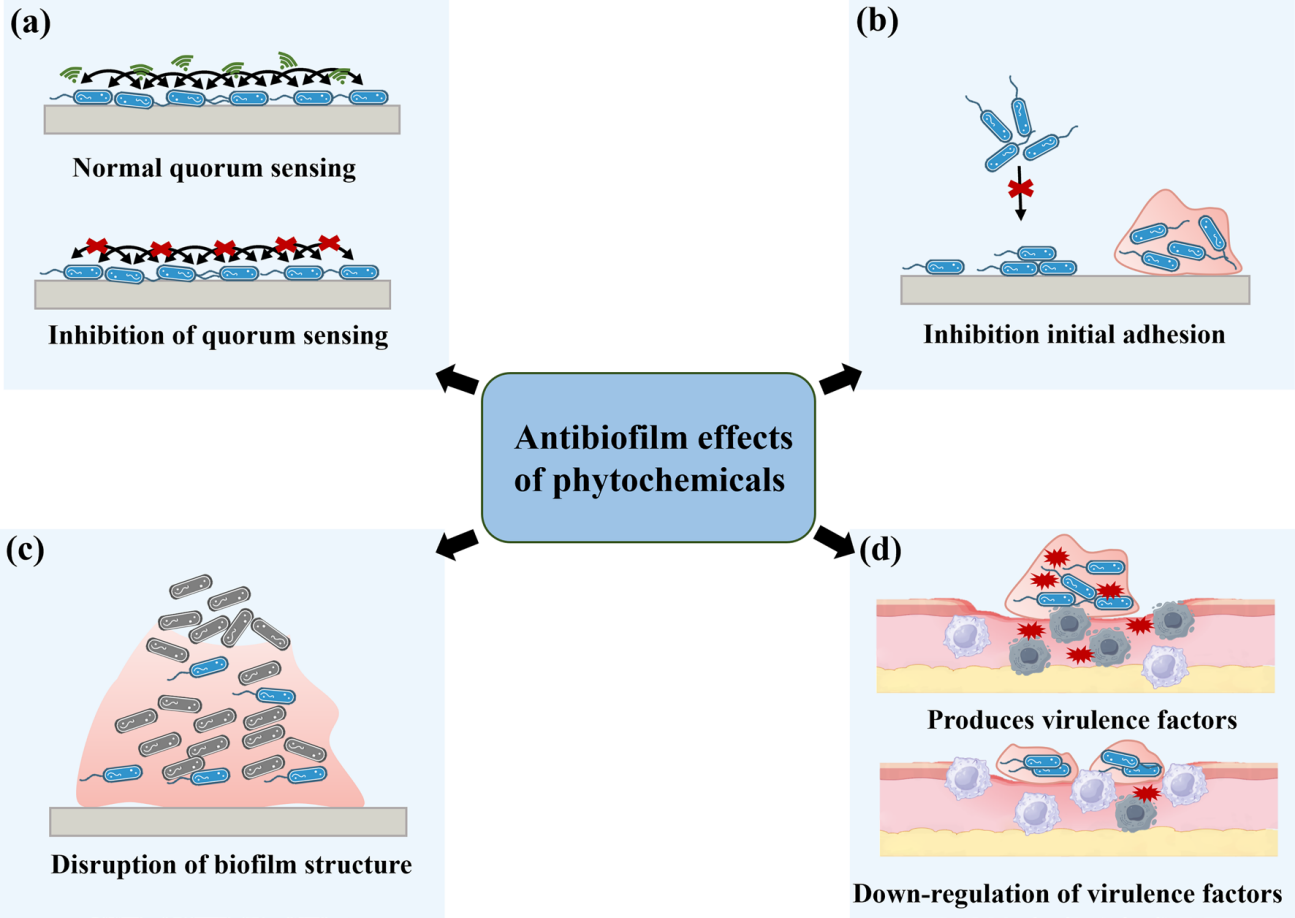
The antibiofilm mechanisms of natural phytochemicals. The antibiofilm mechanisms of natural phytochemicals involve targeting quorum sensing systems, reducing virulence factor production, inhibiting initial adhesion, and disrupting extracellular polymeric substances

Phytochemicals can effectively inhibit involved factors in the growth, maturation, dispersal, and detachment stages of biofilm formation. The associated mechanisms include anti-QS, anti-exopolysaccharide, anti-virulence, and anti-adhesion activities [45]. The distinct mechanisms of action and substantial medicinal value of phytochemicals have positioned them as a focal point for research aimed at developing novel antibiofilm agents. Herein, the most widely studied alkaloids, flavonoids, quinones, non-flavonoid polyphenols, terpenes and others were summarized (Fig. 3), the mechanisms of action and clinical antibiofilm application were summarized (Fig. 4).

### Alkaloids

Alkaloids are the category of nitrogen-containing natural organic compounds that are widely found in plants, such as *Rutaceae* family, *Lauraceae* family, and *Amaryllidaceae* family [46–48]. BBR occurs as an active constituent in the root, rhizome and stem bark of many medicinally important plants, including *Hydrastis canadensis* (goldenseal), *Coptis chinensis* (Coptis or goldenthread), *Berberis aquifolium* (Oregon grape), *Berberis vulgaris* (barberry), and *Berberis aristata* (Tree turmeric) [49]. It can inhibit biofilm formation caused by various bacteria, including *Staphylococcus aureus*, *Streptococcus mutans*, and *P. aeruginosa* [50–52]. Wang et al. found that BBR inhibits Methicillin-resistant *Staphylococcus aureus* (MRSA) biofilm formation by regulating the expression levels of *agrA*, *agrD*, *agrB*, and *agrC*, which in turn affect the expression of extracellular proteins and virulence factors [53]. Autoinducing peptide (AIP) is a signaling molecule involved in quorum sensing, encoded by the *agrD* gene in *Staphylococcus aureus*. Upon secretion and accumulation to a threshold level, AIP binds to the AgrC receptor, which in turn activates the response regulator AgrA. This signaling cascade enhances the expression of RNAIII and other genes associated with virulence, thereby facilitating biofilm formation and the production of toxins [54].

BBR and matrine are reported as QS inhibitors capable of disrupting bacterial communication networks and suppressing biofilm formation [55, 56] BBR interacts directly with the QS signal receptors LasR and RhlR, thereby interfering with the transcriptional regulation of downstream virulence-associated genes and attenuating the pathogenicity of *P. aeruginosa*. At subinhibitory concentrations, BBR can significantly reduce the biofilm biomass of *P. aeruginosa* by 71.70%. In *Escherichia coli*, matrine inhibits biofilm formation by downregulating a series of QS-regulated genes, including luxS and pfS (involved in AI-2 synthesis), sdiA (a LuxR homolog responsive to AHLs), hflX (a GTPase associated with stress response and biofilm maturation), and motA and fliA (critical regulators of flagellar biosynthesis and motility). Study by Yum et al. found that sinomenine exhibited significant inhibitory effects on biofilm formation by *S. aureus*. Sinomenine inhibits intercellular adhesion, polysaccharide intercellular adhesin (PIA), and phenol-soluble modulin peptide production in *S. aureus*, thereby effectively inhibits the biofilm formation and the biofilm dispersion stages [57, 58].

### Flavonoids

Flavonoids are the secondary polyphenolic metabolites commonly found in many fungi and plants [59]. They are a class of C6-C3-C6 compounds consisting of two benzene rings (A-andB-rings) connected by a central three-carbon bond (C-ring) [60]. Based on the oxidation level of the C2 and C3 bonds, the substitution pattern of the B-ring, and whether the three-carbon bond of the C-ring is cyclized, flavonoids are classified into flavones, flavonols, flavanonols, isoflavones, flavan-3-ols and anthocyanins [61]. Wang et al. found that baicalin (BA) inhibits the adhesion and maturation stages of biofilm formation by affecting the production of surface proteins and cell autolysis [62]. In addition, research by Mao et al. found that baicalein could bind to the amino acid residues of LuxS, downregulating the mRNA expression of LuxS and biofilm-related genes, significantly reducing *S. aureus* biofilm formation, and eradicating mature biofilm [63]. Peng et al. confirmed that rutin interferes with QS by reducing the secretion of AI-2 and lowering the expression of virulence genes in avian pathogenic *E. coli* thereby inhibiting biofilm formation [64]. AI-2 synthesized via the LuxS-dependent pathway, serves as a universal QS signal molecule involved in interspecies bacterial communication. AI-2 plays a critical role in modulating bacterial virulence by regulating the expression of QS-associated genes and downstream virulence factors. Moreover, AI-2-mediated quorum sensing is essential for biofilm formation, antibiotic resistance, and immune evasion, all of which contribute to bacterial pathogenicity [65]. Quercetin diminished the production of PIA by downregulating the ica locus to prevent intercellular adhesion in *Staphylococcus epidermidis* [66]. Additionally, quercetin reduced the hydrophobicity of *S. epidermidis*, thereby inhibiting the initial stages of biofilm formation.

### Quinones

Quinones share a structural framework of an *ortho* or *para* substituted dione, which is conjugated either to an aromatic nucleus (benzoquinones) or a condensed polycyclic aromatic system, such as naphthoquinones, anthraquinones, and anthracyclinones [67]. Quinones are also widely distributed in plants, such as *Rheum palmatum* L from the *Polygonaceae* family and *Salvia miltiorrhiza* Bunge from the *Lamiaceae* family. Outer Membrane Protein (OMP) plays a crucial role in the biological membrane formation. It maintains the integrity and stability of the outer membrane as well as participates in regulating the toxicity of bacteria, adapts to the environment, interacts with hosts, and mediates bacterial invasion into host cells [68]. Zhao et al. revealed that aloe-emodin targets OMP6 protein, binds to its regulatory factor ArsR, inhibits the transcription of OMP6 mRNA, disrupts its expression, and suppresses biofilm formation [69]. In addition, aloe-emodin can reduce the accumulation of PIA on the cell surface by inhibiting the production of extracellular proteins and specifically inhibiting the initial adhesion and dispersion stages of *S. aureus* biofilm development [70]. Zu et al. demonstrated that cryptotanshinone possessed significant antibiofilm activity against *S. epidermidis*, with the capacity to eradicate established biofilms [71]. Mechanistically, cryptotanshinone interferes with the structural integrity of biofilms by disrupting the extracellular matrix and altering the overall microarchitecture. At the molecular level, it significantly downregulates the expression of critical biofilm-associated genes, including icaA, atlE, aap and luxS. The gene atlE plays a key role in initiating bacterial adhesion to surfaces, whereas aap facilitates intercellular aggregation, and icaA is responsible for synthesizing PIA, a key structural component of the biofilm matrix. Although these genes do not encode proteins that interact directly, they function synergistically within an integrated regulatory network that orchestrates the establishment, maturation, and structural stability of bacterial biofilms. In addition to impairing gene expression, cryptotanshinone reduces the metabolic activity of bacterial within the biofilm, thereby weakening their viability and resistance.

### Non-flavonoid polyphenols

Phenolic acids, xanthones, stilbenes, lignans, and tannins belong to non‐flavonoid phenolic compounds [72–74]. The term “phenolic acids” generally refers to phenols with one carboxylic acid group. They are derived from two main phenolic compounds, hydroxycinnamic and hydroxybenzoic acids, and it specifically denotes a unique class of organic acids in the context of plant metabolites [75]. Xanthones, stilbenes, lignans, and tannins are compounds with at least two aromatic rings in the structure, whereas only tannins have more aromatic rings. Liu et al. found that, as the concentration of gallic acid increased, the upregulation of the gene icaR repressed the synthesis of PIA [76]. The primary component of polysaccharide slime is PIA, which plays a crucial role in mediating bacterial cell-to-cell adhesion and *S. aureus* biofilm formation. Thus, gallic acid may offer a novel strategy for controlling biofilm-related infections by inhibiting polysaccharide slime formation. Emeka et al. found that mangiferin significantly suppressed the expression of virulence genes in *S. mutans*, suggesting a mechanism involving glucanotransferases, which specifically inhibits colony formation by attenuating bacterial adherence [77]. Moreover, mangiferin significantly suppressed the expression of the gtfB, gtfC, and gtfD virulence genes involved in biofilm formation. He et al. demonstrated that resveratrol at sub-MIC levels significantly reduced biofilm formation in a dose-dependent manner without affecting bacterial growth [78]. Magnolol inhibited *Candida albicans* virulence factors via the PKC and Cek1 MAPK pathways. It significantly reduces adhesion, hyphal formation, biofilm viability, and alters ultrastructure by thinning and rupturing cell walls with cytoplasmic leakage. Moreover, magnolol suppresses the expression of genes related to adhesion, invasion, hyphal formation, biofilm formation, and β-glucan synthesis, including ALS1 and EFG1. Additionally, magnolol downregulated PKC (RHO1, PKC1, BCK1, MKK2, MKC1) and Cek1 pathway-related genes (CDC42, CST20, STE11, HST7, CEK1), suggesting its antifungal effects are mediated through these signaling pathways [79].

### Terpenoids

Terpenoids, also known as isoprenoids, represent one of the most extensive families of natural products, characterized by their diverse structures and wide-ranging biological activities, including monoterpenes (53%), diterpenoids (1%), sesquiterpenes (28%), and others (18%). The core structural unit of terpenes is the isoprene unit (C_5_H_8_), chemically identified as “2-methyl-1,3-butadiene,” which is a branched, unsaturated hydrocarbon [80]. Yuan et al. found that thymol could inhibit biofilm formation by inhibiting the production of PIA and the release of eDNA and could eradicate already formed biofilms [81]. eDNA is an essential component for biofilm formation, as it plays key roles in bacterial adhesion, aggregation, microcolony formation and biofilm architecture [82]. In the mouse infection model, the combining treatment with thymol and vancomycin significantly enhanced the eradication of MRSA biofilms and alleviated inflammation. Andrographolide is a QS inhibitor that reduces monocyte proliferation by targeting the QS system. It significantly decreases the expression levels of the agr gene and the activity of the agr promoter P2, effectively eradicating biofilms [83]. Besides, andrographolide sulfonate has been shown to inhibit biofilm formation and enhance biofilm permeability in MRSA [84]. These effects are primarily mediated through the suppression of key gene expressions involved in biofilm regulation. Specifically, Andrographolide sulfonate downregulates quorum sensing regulatory genes (agrD and sarA), microbial surface component-recognizing adhesion matrix genes (clfA and fnbB), intercellular adhesion-associated genes (icaA, icaD, and PIA), and a gene implicated in eDNA release (cidA). In addition to genetic modulation, Andrographolide sulfonate also reduces the levels of five biofilm-related metabolites, including anthranilic acid, D-lactic acid, kynurenine, L-homocitrulline, and sebacic acid, further contributing to the disruption of biofilm integrity. Anthranilic acid plays a crucial role during the initial stages of biofilm formation by enhancing bacterial adherence to abiotic surfaces and modulating the transcription of EPS-related operons essential for matrix production [85]. Andrographolide sulfonate disrupts biofilm formation in Staphylococcus aureus by suppressing anthranilic acid synthesis, thereby impairing cell adhesion and EPS biosynthesis. D-lactic acid, a fermentation-derived metabolite, serves as an electron donor in the aerobic regions of biofilms, supporting metabolic activity and energy generation in stratified microbial communities [86]. The reduction of D-LA levels following Andrographolide sulfonate treatment may impair energy metabolism in sessile MRSA cells, thereby limiting biofilm growth and compromising structural integrity. Additionally, phenylpyruvic acid, D-2-aminobutyric acid, and L-tyrosine are recognized as key metabolites that are enriched during MRSA biofilm development [84]. The Andrographolide sulfonate-induced suppression of these metabolites may interfere with essential metabolic pathways necessary for biofilm homeostasis and persistence.

### Others

Cinnamaldehyde, which is a member of the aldehyde class, is a naturally occurring compound commonly found in the bark of cinnamon trees. He et al. reported that cinnamaldehyde inhibits the *S. mutans* biofilm formation in a dose-dependent manner [87]. This inhibition mechanism mainly reduces bacterial aggregation by increasing the hydrophobicity of the bacterial surface and reducing adhesion. In addition, cinnamaldehyde regulates the expression of biofilm toxicity genes and reduces biofilm formation and microbial metabolic activity. Mechanisms of action of phytochemicals against biofilms are shown in Tables 1 and 2.

**Table 1:** Mechanisms of action of phytochemicals against biofilms

No	Components	Structural formula	Category	Main source	Bacteria	MIC/MBC; MBIC/MBEC	Antibiofilm mechanism	Reference
			**Alkaloid**					
**1**	Berberine		Isoquinoline	*Coptis chinensis* Franch	*Pseudomonas aeruginosa* PAO1	MIC = 0.5 µg/ml	Inhibiting biofilm efflux pump PA MexXY-OprM	[51]
**2**	Hordenine		Phenethylamine	*Hordeum vulgare* L	*Pseudomonas aeruginosa*	MIC = 2.5 mg/ml	Down regulating QS related genes lasI, lasR, rhlI and rhlR; Inhibiting of acyl homoserine lactones	[88]
**3**	Matrine		Quinolizidine	*Sophora flavescens* Aiton	*Escherichia coli*	MIC = 5.12 mg/ml	Down regulating of QS related genes luxS, pfS, sdiA, hflX, motA and fliA	[50]
**4**	Sinomenine		Tetracyclic morphinan	*Sinomenium acutum (Thunb.) Rehder& E. H. Wilson*	*Staphylococcus aureus*	MBIC = 0.1 µM	Up-regulating agrA and down-regulating icaA gene expression	[57]
			**Flavonoids**					
**5**	Baicalein		Flavone	*Scutellaria baicalensis* Georgi	*Staphylococcus aureus*	MBIC = 1024 µg/ml	decreasing AI-2 activity and luxS expression. QS inhibitors for LuxS/AI-2 systems	[63]
**6**	Hesperidin		Flavanone glucosyl	*Citrus reticulata* Blanco	Methicillin-resistant *Staphylococcus aureus*	MBIC = 100 µg/ml	Downregulating the expression of sarA, icaA, icaD, altA, fnbA, fnbB, and crtM genes	[89]
**7**	Hyperoside		Flavonol glycoside	*Hypericum monogynum* L	*Pseudomonas aeruginosa*	MBIC = 16 µg/ml	Inhibiting of lasR, lasI, rhlR and rhlI gene expression	[90]
**8**	Kaempferol		Flavonol	*Kaempferia galanga* L	*Staphylococcus aureus*	MBIC = 64 µg/ml	Inhibiting the expression of SrtA, ClfA, ClfB, FnbpA, and FnbpB	[91]
**9**	Myricetin		Flavonol	*Morella rubra* Lour	*Streptococcus mutans*	MIC = 512 ug/ml	Inhibiting SrtA activity and release Pac	[92]
**10**	Naringin		Flavanone glucosyl	*Citrus reticulata* Blanco	Metallo-β-lactamases (MβLs) *Pseudomonas*	MIC = 128 µg/ml	Decreasing EPS and alginate	[93]
**11**	Phloretin		Dihydrochalcone	*Malus pumila* Mill	*Serratia marcescens*	MIC > 2 mg/ml	Inhibiting of AHLs secretion reducing protease and EPS production	[94]
**12**	Quercetin		Flavonol	*Quercus dentata* Thunb	*Staphylococcus epidermidis*	MIC = 125 µg/ml	Reducing the production of PIA; Changing EPS components	[66]
**13**	Tea catechin epigallocatechin gallate		Flavan-3-ols	*Camellia sinensis* (L.) Kuntze	*Porphyromonas gingivalis*	MBC = 0.5 mg/ml	Inhibiting of *P.gingivalis* initial adhesion stage	[95]
**14**	Rutin		Flavonol glucosyl	*Styphnolobium japonicum* (L.) Schott	Avian Pathogenic *Escherichia coli*	MIC > 50 µg/ml	Reducing AI-2 generation and interfere with QS system	[64]
**15**	Wogonin		Flavone	*Scutellaria baicalensis* Georgi	*Pseudomonas aeruginosa* PAO1	MIC > 30 µg/ml	Down-regulating the expression of QS related genes and reducing the production of virulence factors such as elastase, pyocyanin and proteolytic enzyme	[96]
			**Quinones**					
**16**	Aloe-emodin		Anthraquinone	*Rheum palmatum* L	*Staphylococcus aureus*	MBIC = 64 µg/ml	Combining with ArsR to target the inhibition of OMP6 mRNA expression	[69]
**17**	Cryptotanshinone		Phenanthrenequinones	*Salvia miltiorrhiza* Bunge	*Staphylococcus epidermidis*	MIC = 2 µg/ml MBIC = 32 µg/ml	Down-regulating expression of icaA, atlE, aap and luxS	[71]
			**Terpenoids**					
**18**	Andrographolide		Diterpenoid	*Andrographis paniculata* (Burm. f.) Nees	Methicillin-resistant *Staphylococcus aureus*	MIC = 50 µg/ml	Inhibiting of icaD, fnbB and PIA gene expression; sarA and agrD were down-regulated	[84]
**19**	Carnosol		Diterpenoid	*Salvia japonica* Thunb	*Candida albicans*	MIC = 0.1 mg/ml	Inhibiting the transformation of yeast into mycelium	[97]
**20**	Carvacrol		Monoterpenoid	*Thymus mongolicus* Ronniger	*Chromobacterium violaceum*	MBIC = 0.1 mM	Down-regulating cviI expression, purple pigment production and chitinase activity	[98]
**21**	Geraniol		Acyclic monoterpene	*Rosa rugosa* Thunb	Methicillin-resistant *Staphylococcus aureus*	MIC = 512 µg/ml MBC = 1024 µg/ml	Reducing the release of extracellular nucleic acid eDNA and inhibit cellular autolysis	[99]
**22**	Ginsenoside Rh2		Triterpene saponin	*Panax ginseng* C. A. Mey	*Streptococcus mutans*	MIC_50_ = 3.48 ng/μl	Reducing EPS production, down-regulated mannose-specific IIC/D components and acetaldehyde/alcohol dehydrogenase	[100]
**23**	Menthol		Monoterpenes	*Mentha canadensis* L	*Candida albicans*	MIC_50_ = 3.125 µg/ml	Downregulating ALS1, ALS3, and HWP 1	[101]
**24**	Nootkatone		Sesquiterpenes	*Citrus maxima* (Burm.) Merr	Methicillin-resistant *Staphylococcus aureus*	MIC = 101.6 µg/ml	Inhibiting of NorA, Tet(K), MsrA,andMepA proteins bacterial efflux pump	[102]
**25**	Thymol		Monoterpenes	*Trigonella foenum-graecum* L	Methicillin-resistant *Staphylococcus aureus*	MIC = 256 µg/ml	Inhibiting of PIA production and eDNA release; TNF-α and IL-6	[81]
			**Non-flavonoid polyphenols**					
**26**	Chlorogenic Acid		Phenylpropionic acid	*Eucommia ulmoides* Oliv	*Pseudomonas aeruginosa*	MIC = 25.4 mM	Interferencing with the synthesis and transcription of signaling molecules modulates the Las, pqs, and Rhl systems, and attenuates β-alanine metabolism and pyrimidine metabolism	[103]
**27**	Curcumin		Diarylterpenoid	*Curcuma longa* L	*Pseudomonas aeruginosa*	MIC = 200 µg/ml	Suppressing LasI/LasR system and QS process of LuxS/ AI-2 system	[104]
**28**	Gallic acid		Phenolic acid	*Rhus chinensis* Mill	*Staphylococcus aureus*	MIC = 2 mg/ml MBC = 8 mg/ml	Up-regulating icaR, down-regulated icaA and icaD	[76]
**29**	Magnolol		Lignan	*Houpoea officinalis* (Rehder& E. H. Wilson) N. H. Xia & C. Y. Wu	*Candida albicans*	MBIC = 160 µg/ml	Down-regulating PKC pathway-related genes and Cek1 pathway-related genes	[79]
**30**	Mangiferin		Xanthone glycoside	*Anemarrhenaasphodeloides* Bunge	*Streptococcus mutans*	MIC = 500 µM	Down-regulating the expression of gtfB, gtfC and gtfD virulence genes involved in biofilm formation	[77]
**31**	Punicalagin		Ellagitannin	*Punica granatum* L	*Staphylococcus aureus*	MIC = 0.25 mg/ml	K^+^ outflow, plasma membrane morphology changed	[105]
**32**	Resveratrol		Stilbens	*Reynoutria japonica* Houtt	*Fusobacterium nucleatum*	MIC = 100 µg/ml	Promoting bacterial aggregation and reduce BF formation; Down-regulating the expression of QS related genes FN0116, FN0503, FN0659, FN0675 and FN1856	[78]
**33**	Rosmarinic acid		Phenylpropionic acid	*Rosmarinus officinalis* L	*Candida albicans*	MIC = 0.1 mg/ml; MBIC = 0.4 mg/ml	Decreasing mitochondrial activity, changes in exopolysaccharide, membrane integrity and inhibiting of protease hydrolase production	[106]
**34**	Shikimic acid		Cyclohexanecarboxylic acid	*Illicium lanceolatum* A. C. Sm	*Staphylococcus aureus*	MIC = 1.25 mg/ml	Down-regulating sarA gene transcription and up-regulating agrA gene transcription	[107]
			**Others**					
**35**	Allicin		Thioester	*Allium sativum* L	Uropathogenic *Escherichia coli*	MIC > 200 µg/ml	Down-regulating the expression of the TCS homologous response regulatory gene uvrY, and increasing the expression of the RNA-binding global regulatory protein gene csrA associated with UPEC biofilm	[108]
**36**	Cinnamaldehyde		Aldehyde	*Cinnamomum cassia* (L.) D. Don	*Streptococcus mutans*	MIC = 1 mg/ml MBC = 2 mg/ml	Down-regulating the expression of sarA gene	[87]
**37**	Eugenol		Phenylpropenes	*Eugenia caryophyllata* Thunb	Methicillin-resistant *Staphylococcus aureus*	MIC = 3.125% ~ 0.01%	Decreasing the expression levels of IcaA, IcaD and SarA	[109]
**38**	Sulforaphane		Isothiocyanates	*Brassica oleracea* var*. italica* Plenck	*Streptococcus* *mutans*	MIC = 256 µg/mL	Suppressing the synthesis of EPS and acid production	[110]

*MIC*: Minimum inhibitory concentration. *MBC*: Minimum bactericidal concentration. *MBIC*: Minimum biofilm formation inhibitory concentration. *MBEC*: Minimum biofilm eradication concentration

**Table 2:** Phytochemicals categorized by mechanistic targets in antibiofilm activity

Mechanistic Target	Phytochemical Class	Representative Compounds	Mechanism of Action	Typical Targets/Pathways	Reference
Quorum sensing inhibition	Alkaloid, Flavonoids, Quinones, Terpenoids, Non-flavonoid polyphenols	Hordenine, Matrine, Sinomenine, Baicalein, Hesperidin, Hyperoside, Kaempferol, Rutin, Wogonin, Cryptotanshinone, Menthol, Chlorogenic Acid, Curcumin, Resveratrol, Shikimic acid, Cinnamaldehyde, Eugenol	Down regulating QS related genes; Inhibiting of acyl homoserine lactones	LasR, RhlR, LuxS, agrA, sar	[56, 57, 63, 64, 70, 71, 87–92, 96, 101, 103, 104, 107, 109]
EPS matrix degradation	Flavonoids, Terpenoids, Non-flavonoid polyphenols	Naringin, Phloretin, Quercetin, Geraniol, Ginsenoside Rh2, Thymol, Gallic acid, Rosmarinic acid	Decreasing EPS, eDNA, protease and alginate	EPS, pel, psl, alg operons	[76, 81, 93, 94, 99, 100, 106, 110]
Surface adhesion inhibition	Flavonoids, Terpenoids, Non-flavonoid polyphenols	Quercetin, Tea catechin epigallocatechin gallate, Andrographolide, Thymol, Gallic acid	Reducing the production of PIA;	Surface proteins, ica	[66, 76, 81, 84, 95]
Signal transduction pathway interference	Non-flavonoid polyphenols	Magnolol	Down-regulating PKC pathway-related genes and Cek1 pathway-related genes	PKC pathway, Cek1 pathway, MAPK,	[79]
Efflux pump inhibition	Alkaloid, Quinones, Terpenoids	Berberine, Aloe-emodin, Nootkatone	Inhibiting biofilm efflux pump PA MexXY-OprM; inhibition of OMP6 mRNA expression	PA MexXY-OprM; NorA, Tet(K), MsrA, andMepA	[51, 69, 102]
Reduction of virulence factor expression	Flavonoids, Terpenoids, Non-flavonoid polyphenols	Wogonin, Carvacrol, Carnosol, Mangiferin, Rosmarinic acid, Shikimic acid, Eugenol	reducing the production of virulence factors	agrA, hla, spa, sigB, saeRS, sar	[77, 96–98, 106, 107, 109]

### Structure–activity relationships of phytochemicals with antibiofilm potential

To further advance research in this area, increasing attention has been directed toward elucidating the structure–activity relationships (SAR) of phytochemicals. Flavonoids play an important role in their antibacterial properties affecting by its amphipathic features [111]. Anthraquinone skeleton appear to play a crucial role in inhibiting biofilm formation at low concentrations, in which the hydroxyl groups at the C-1 and C-2 positions play vital role [112]. Besides, quinones exert antibacterial effects by generating reactive free radicals, forming irreversible complexes with essential proteins, and disrupting cellular structures and metabolic pathways [113, 114]. And there is also evidence shows that berberine (alkaloid) is a bioactive group that binds to DNA molecules to produce antibacterial effects [115]. In addition to the influence of core skeletal structures on antibacterial properties, the type, position, and number of substituent groups play a critical role in determining the antibiofilm activity of phytochemicals. These variations can significantly alter key molecular characteristics such as hydrophobicity (e.g., through alkyl or terpene chains), lipophilicity (facilitating penetration of bacterial lipid membranes), planarity (as seen in aromatic systems that enable intercalation into nucleic acids), and charge distribution (e.g., the presence of cationic moieties enhancing electrostatic interaction with negatively charged bacterial surfaces) [114–118]. It should be note that comparing with the research on the SAR of antibacterial properties of phytochemicals, the research on the SAR of their antibiofilm is still in its infancy. Base on the recent findings, key structures features that are closely associated with antibiofilm activity were summarized as following:

(i) The position and number of hydroxyl groups significantly influence the antibacterial activity of flavonoids. For example, the combination of 5,7-dihydroxyl groups on the A-ring and a 4'-hydroxyl group on the B-ring constitutes a pharmacophore associated with high activity, whereas methylation of hydroxyl groups tends to reduce antibiofilm efficacy [119–121].
(ii) Hydrophobic substituents (e.g., terpene chains, alkyl chains, and aromatic groups) generally enhance the ability of compounds to penetrate bacterial and biofilm membranes, thereby improving their antibacterial and antibiofilm activity (e.g., 8-hydroxyquinoline and polyphenols) [114, 122, 123].
(iii) Positively charged structural moieties (e.g., quaternary ammonium ions and cationic alkaloids) facilitate electrostatic interactions with negatively charged bacterial membranes and enhance intracellular accumulation, thereby helping to overcome biofilm barriers and improve bactericidal efficacy, as exemplified by berberine [119, 120].
(iv) Metal ion chelation is a key antibacterial mechanism for certain structures (e.g., 8-hydroxyquinoline and polyphenols), acting through sequestration of essential metal cofactors required for biofilm formation or inhibition of metalloenzyme targets [124, 125].

## Antibiofilm applications of natural phytochemicals in clinic

**Fig. 5:**
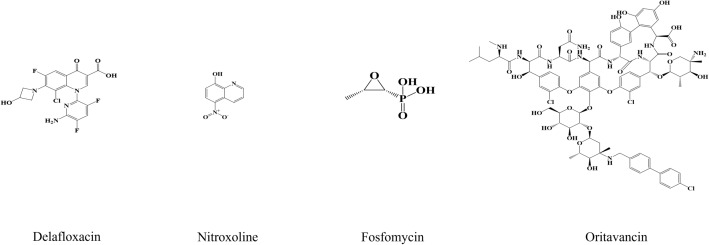
Chemical structures of clinically used antibiofilm agents. Several FDA-approved antibiotics have shown notable antibiofilm activity in both preclinical and clinical studies. These agents are particularly effective in managing biofilm-associated infections related to indwelling medical devices, chronic wounds, and respiratory tract biofilms

Several FDA-approved antibiotics have demonstrated significant antibiofilm effects in preclinical and clinical studies, particularly in infections involving indwelling medical devices, chronic wounds, and respiratory biofilms. We have included representative chemical structures of some key agents (Fig. 5) with reported antibiofilm activity: Delafloxacin: A fluoroquinolone with an anionic character, enhances accumulation in acidic biofilm environments. Nitroxoline: A hydroxyquinoline derivative; chelates Fe^2+^ and Zn^2+^ from the biofilm matrix. Fosfomycin: A phosphonic acid derivative; inhibits early cell wall synthesis and has been shown to penetrate biofilms. Oritavancin: A lipoglycopeptide with both anti-cell wall and membrane-disrupting activity, useful in biofilm-associated Gram-positive infections. Despite the demonstrated antibiofilm potential of certain antibiotics, their clinical efficacy remains limited due to the adaptive resistance, which are caused by limited antibiotic diffusion, enhanced tolerance to antibiotics through persister cell formation, QS-mediated regulation of genes associated with biofilm maturation and resistance, efflux pump upregulation, and enzyme-mediated drug degradation [21–23, 28].

The above limitations have prompted increasing interest in combination strategies, particularly those involving natural phytochemicals. Carvacrol and thymol exhibited a synergistic effect against a methicillin-susceptible Staphylococcus aureus (MSSA) strain (FICI = 0.50). Furthermore, when combined with tetracycline or chloramphenicol, carvacrol and thymol demonstrated significant synergistic activity against both MSSA and MRSA strains, with FICI values ranging from 0.25 to 0.50 [126]. Some combinations achieved more than 50% biofilm reduction at both MICs and sub-MICs [126]. Cuminaldehyde and gentamicin exhibited synergistic antibacterial effects (FICI = 0.36) and significant antibiofilm activity against *S. aureus* [127]. In addition, the combined use of allicin at a concentration of 4 mg/L and vancomycin could inhibit the biofilm formation of *S. epidermidis* on artificial joints [128]. Chadha et al. revealed that the combination of α-T and GeN exhibited strong synergistic activity against *P. aeruginosa* PAO1, as indicated by significant reductions in their MICs (16-fold for α-T and eightfold for GeN) and a low FICI index of 0.1875 [129]. These synergistic effects are primarily achieved through multiple mechanisms of action, including membrane disruption, quorum sensing inhibition, suppression of biofilm formation, induction of oxidative stress, inhibition of efflux pumps, and interference with essential metabolic and resistance pathways [127, 130]. As a result, antibiotic–phytochemical combination therapy offers enhanced antibacterial efficacy with reduced dosages, improved penetration into biofilms, inhibition of resistance mechanisms, delayed emergence of antimicrobial resistance, and broad-spectrum synergistic potential [129, 131, 132]

In addition to their synergistic use with antibiotics, emerging evidence also highlights the promising standalone efficacy of phytochemicals in the clinical management of biofilm-associated infections. Clinical evidence of the therapeutic effects of natural phytochemicals in the treatment of biofilm-related infections would provide deeper insights for future research and applications. De Araújo et al. grouped 36 patients with oral candidiasis to evaluate the efficacy of cinnamon essential oil mouthwash in the treatment of oral candidiasis [133]. According to the colony-forming unit results, the use of cinnamon essential oil mouthwash reduced the number of Candida spp. in the oral mucosa by 61%. Gabrielian et al. conducted a double-blind randomised placebo-controlled trial involving 185 patients with upper respiratory tract infections [134]. The results showed that individuals treated with verum exhibited the most significant improvement in headache, nasal and throat symptoms, and overall malaise compared to the placebo group. This study suggests that verum has beneficial effects in the treatment of acute upper respiratory tract infections and can alleviate sinusitis symptoms. A total of 139 patients with knee osteoarthritis participated in this randomised study and were divided into two groups: one group received 500 mg of turmeric capsules three times daily, while the other group received 50 mg of diclofenac tablets twice daily for 28 days. On days 14 and 28, patients receiving turmeric treatment showed similar improvements in pain severity and KOOS scores as those receiving diclofenac treatment [135]. The clinical applications of natural phytochemicals in the treatment of biofilm-related infections are detailed in Table 3.

**Table 3:** Treatment of biofilm-related clinical diseases with phytochemicals

No	Natural products	Source plant	Trial type	Disease	Number of patience	Control drug	Test time	Observation index	Reference
1	Cinnamaldehyde	*Cinnamomum cassia* (L.) D. Don	Random and double blind	Oral candidiasis	36	Nystatin	15 D	Candida decreased by 61%	[133]
2	Andrographis paniculata	*Andrographis paniculata* (Burm. f.) Wall. ex Nees	Double blind	Nasosinusitis	185	Placebo	5 D	Headache, throat, nose relief	[134]
3	Curcumin	*Curcuma longa* L	Random and double blind	Knee osteoarthritis	139	Diclofenic acid	28 D	The pain level was similar to KOOS and control group	[135]
4	Sinomenine	*Sinomeniumacutum (Thunb.) Rehder& E. H. Wilson*	Random parallel	Rheumatoid arthritis	101	Leflunomide	24 W	Inflammation markers ESR and CRP decreased	[136]
5	Centiderm	*Centellaasiatica* (L.) Urb	Random parallel	Local burn	75	Silver sulfadiazine	14 D	Reepithelialization, wound healing	[137]

## Natural phytochemical-based nanoparticles for biofilm-infection treatment

Nanoparticles (NPs) with the particle size in the range of 1–100 nm [138] have specific physical and chemical characteristics, such as optical, magnetic, and electrical properties. Notably, the physicochemical characteristics of nanomaterials can be precisely controlled by adjusting their size, shape, and composition [139]. Among them, NP size as a crucial factor, in fighting biofilm-related infections can enhance permeability and retention effect, allow sustained release and realize target delivery to enhance efficacy and reduce side effects [140, 141]. What is more, the surface modification of nanomaterials influences the interactions between biofilm and nanomaterials (e.g., mechanical disruption, electron transfer, enzymatic degradation, oxidative stress, and hyperthermia), which further imparts multifunctional capabilities, such as targeted delivery, environmental responsiveness, and controlled release [142].

Natural phytochemical-based nanotechnology involves processing the natural phytochemicals using various nanotechnologies, through self-assembly, encapsulation, emulsification, or surface functionalization to transform natural phytochemicals into NPs with distinct physicochemical properties. Natural phytochemical-based nanocarriers have distinct advantages: (i) Some natural phytochemicals can self-assemble into NPs, with drug loading efficiency as high as 100% [143]; (ii) Natural phytochemicals possess excellent biocompatibility and controllable degradability, thereby minimizing side effects and immunogenicity [144]; (iii) Natural phytochemicals derived from natural products exhibit strong pharmacological activity and low biological toxicity, and (iv) Natural phytochemicals are renewable and environmentally friendly [145].

**Fig. 6:**
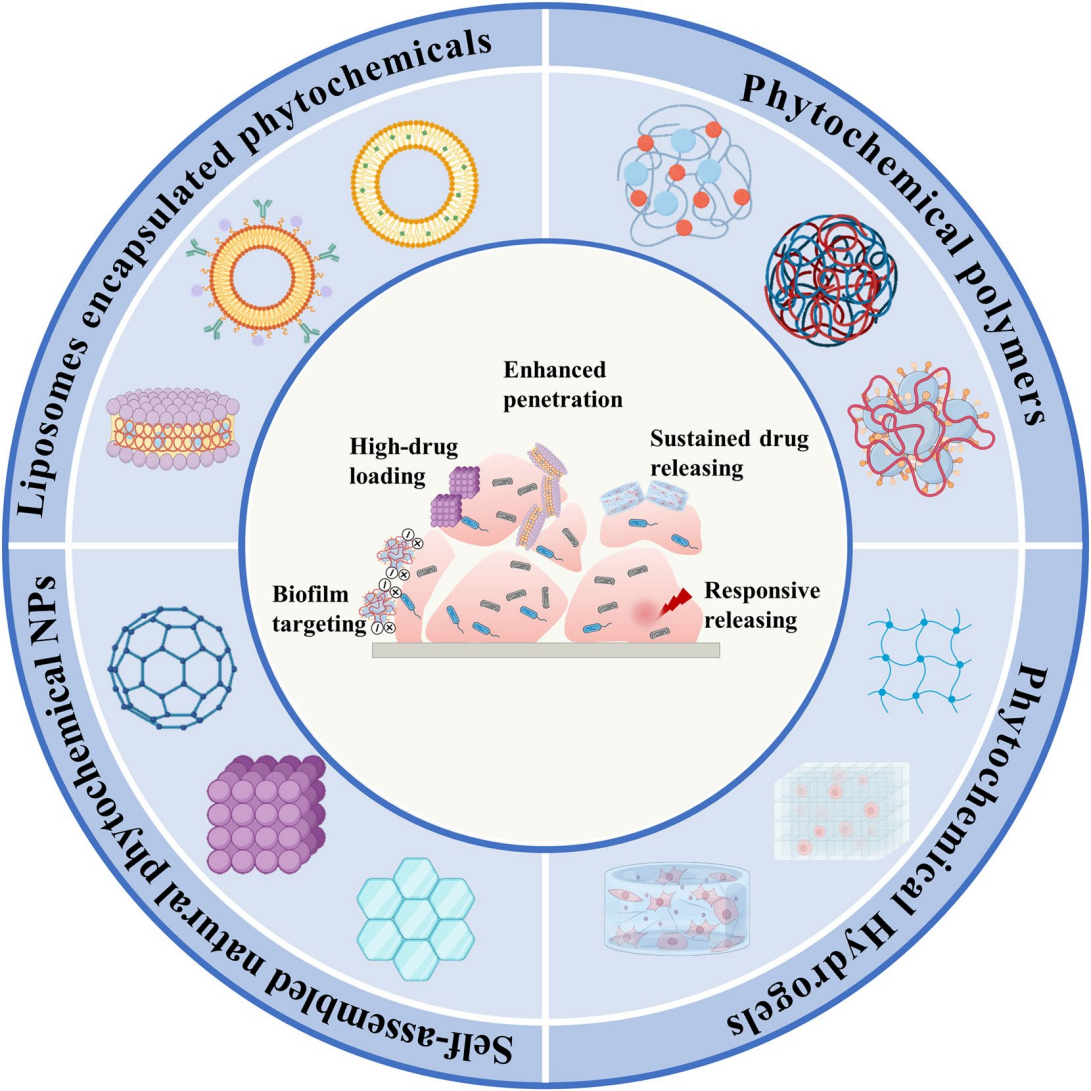
The improved antibiofilm mechanisms of phytochemical-based NPs. Functionalization of phytochemicals by self-assembly, liposome encapsulation, polymerization and hydrogelation to enhance antibiofilm effects. They are characterized by high drug loading and permeability, as well as the ability to target biofilms, sustain prolonged drug delivery and responsive drug release

Overall, the properties of natural phytochemicals can be effectively combined with the advantages of nanostructures in the design of advanced nanomedicines. This review focuses on natural phytochemical-based nanomaterials with characteristic properties on antibiofilm (Fig. 6), laying the foundation for the future development and clinical translation of effective antibiofilm therapies.

### Self-assembled natural phytochemical NPs

Self-assembly refers to the spontaneous formation of ordered structures from disordered fundamental components driven by non-covalent interactions [146]. Compounds under supramolecular driving forces usually have structures capable of multiple non-covalent interactions, including hydrogen bonding, π-π packing interaction, electrostatic attraction, van der Waals force, and hydrophobic interaction [147]. Current research indicates that terpenoids, alkaloids, flavonoids, quinones, and polyphenols are representative compounds capable of self-assembly. These similar structures exhibit comparable self-assembly characteristics, facilitating self-assembly process evaluation [148]. Several studies suggest that self-assembled products outperform single molecules in pharmacological activity. Besides, self-assembly can enhance antibiofilm efficacy through synergistic effects, reduced toxicity, and improved transmembrane capacity [149]. Tian et al. used herb wormwood as a raw material to prepare Berberine-Rhein (BBR-Rhe) NPs with antibacterial activity through self-assembly [150]. The removal rate of *S. aureus* biofilms by BBR-Rhe NPs significantly improved by 50.8%. In addition, BBR-Rhe NPs adhered to the surface of the bacteria and increased the concentration of drugs around them, causing severe damage to the integrity of *S. aureus*, ultimately leading to its death. Xie et al. used Fmoc-phenylalanine (Fmoc-F) and BBR to self-assemble a biocompatible hybrid hydrogel (Fmoc-F/BBR hydrogel) with aggregation-induced emission (AIE) characteristic [151]. This hydrogel drove the aggregation of BBR through intermolecular electrostatic interactions and π-π stacking, performing AIE-active property. BBR NPs dispersing throughout the amino acid nanofiber hydrogel, can effectively generate reactive oxygen species (ROS), thereby disrupting the biofilm structure and function. Under white light irradiation, the Fmoc-F/BBR hydrogel demonstrated potent antibacterial activity against *E. coli* and *S. aureus*. Moreover, the research confirmed its ability to eradicate already formed biofilms and kill bacteria, with low bacterial toxicity and haemolytic activity.

### Functionalized natural phytochemical NPs

Functionalization allows the introduction of specific reactive groups, enabling precise targeting, controlled release, and environmental responsiveness [152]. For example, conjugation with polymers or surfactants improves the solubility and bioavailability of the phytochemicals; hybridization with liposomes or hydrogels further endows the phytochemicals with structural flexibility, prolonged stability, and multifunctional capabilities. These advancements not only expand the utility of phytochemicals in drug delivery and tissue engineering but also align with the increasing demand for sustainable and eco-friendly materials. Through these approaches, phytochemicals transcend their traditional roles and emerge as versatile components in innovative material systems.

### Liposomes encapsulated phytochemicals

As a formulation for loading hydrophobic drugs, liposomes are currently in mature development, with their phospholipid bilayer structure allowing for more effective interactions with membranes and penetration into their interiors. Liposome encapsulation can significantly enhance the efficacy of antibiofilm while maintaining drug activity, thereby disrupting the structure of biofilms or inhibiting microbial growth [153]. Plant chemicals such as BBR and Cur have remarkable antibiofilm activity. However, their efficacy is often limited by their low bioavailability, poor water solubility, and poor cell permeability. When being co-encapsulated into liposomes, the minimum inhibitory concentrations of BBR and Cur were reduced by 87% and 96%, respectively. The fractional inhibitory concentration of 0.13 indicated synergistic effect. Comparing with free drugs, BBR and Cur liposomes are more effective in suppressing MRSA growth and preventing biofilm formation [154]. Garlic treatment of biofilms can reduce the production of virulence factors and QS signaling [155]. However, garlic alone is insufficient to eradicate biofilms in cystic fibrosis. Ghodake et al. demonstrated that garlic liposomes can directly fuse with the outer membrane of *P. aeruginosa* and release the active garlic extract into the biofilms, directly kill bacteria within the biofilms and significantly increase the antibacterial and antibiofilm activity against *P. aeruginosa* [156]*.*

### Phytochemical polymers

The formulation of phytochemicals into polymer NPs (e.g., micelles, vesicles, star polymers) of various shapes and sizes offers significant advantages over linear polymers for specific applications, including drug and gene delivery. One of the main advantages is the multivalency of polymeric NPs, Nanoparticles with a cluster of multiple functional groups can enhance the cellular interactions [157]. Zhang et al. used β-cyclodextrin grafted chitosan (CDCS) as a drug carrier to prepare baicalin-β-cyclodextrin grafted chitosan NPs (CDCS-BA NPs) to enhance the antibiofilm efficacy of BA [158]. CDCS-BA NPs with positively charged surface are able to rapidly adhere to the negatively charged biofilms, and BA is released from the lipophilic cavity of the CDCS to reach the interior of the biofilms, effectively killing bacteria. Thus, the biofilm growth was prevented over time and the biofilm formation was effectively inhibited. Subhaswaraj et al. encapsulated cinnamaldehyde into chitosan NPs, the slow and sustained release of cinnamaldehyde from cinnamaldehyde NPs indicated its persistence as an effective QS inhibitor, whose mechanism is related to the Las, Rhl, and pqs systems [159]. Cinnamaldehyde NPs inhibit biofilm formation by down-regulating the virulence factor pyocyanin and reducing LasA protease activity. Furthermore, Cinnamaldehyde NPs of sub-MIC concentrations significantly affected the motility and swarming of *P. aeruginosa*, further inhibiting the occurrence of bacterial group reactions.

### Phytochemical hydrogels

Hydrogels are 3D network materials structures composed of natural or synthetic hydrophilic polymers. Hydrogels can impart NPs with properties such as sustained drug release, theranostic capability, as well as conductivity and adhesiveness, making them highly valuable for antibiofilm and wound dressings [160]. Wang et al. designed chitosan-based nanogels to encapsulate Tanshinone IIA (TA) to enhance their antibacterial and antibiofilm effects against *S. mutans* [161]. Under illumination, the stability of TA in the hydrogel was significantly improved and its antibacterial activity was enhanced by at least fourfold. Furthermore, Tanshinone IIA@Chitosan nanogels (TA@CS) exhibited pH responsiveness, enabling the selective release of more TA under acidic conditions. The positively charged TA@CS targeted the negatively charged surface of the biofilms and effectively penetrated the biofilm, thus demonstrating more pronounced antibiofilm activity. Hu et al. encapsulated BBR into natural microalgae Spirulina (SP) and combined it with carboxymethyl chitosan/sodium alginate to create a bioactive hydrogel, BBR-encapsulated SP hydrogel (BBR@SP gel) [162]. Under laser irradiation, the gel released BBR and generated ROS, achieving synergistic QS inhibition and chemo-photodynamic therapy against MRSA. Additionally, the gel inhibited biofilm formation, disrupted existing biofilms, and downregulated virulence factors. Finally, BBR@SP gel accelerated MRSA-infected diabetic wound healing by promoting angiogenesis, enhancing skin regeneration, and reducing inflammation.

## Conclusions and future perspectives

This work offers an in-depth review on the advancements of natural phytochemicals in antibiofilm applications over the past decade. A total of 38 natural phytochemicals, mainly including alkaloids, flavonoids (e.g., flavonols, flavanols, and chalcones), quinones, non-flavonoid polyphenols, terpenes are discussed in detail. Natural phytochemicals can inhibit biofilm formation at its initial stages by suppressing QS systems and reducing the production of PIA and EPS, thus mitigating healthcare-associated infections. Inhibition of QS appears to be the main mechanism of action for many natural phytochemicals, as they prevent the accumulation of QS signal molecules within biofilms, thereby downregulating the expression of virulence factors, and attenuating the immune response of the organism. Furthermore, these natural phytochemicals are also capable of eradicating mature biofilms by inhibiting the expression of key genes, such as QS genes (e.g., luxS), adhesion genes (e.g., fnbA), and protease secretion, thereby disrupting the structural integrity of the biofilm.

Phytochemical-based nanomaterials, via modifications such as self-assembly, liposome encapsulation, polymerization, and hydrogelation, enhance drug loading, targeted delivery, and sustained release. These nanocarriers improve the bioavailability and biofilm penetration of phytochemicals, simultaneously protecting them from environmental degradation. The small size and customizable surface properties of nanomaterials facilitate enhanced tissue penetration and specific targeting of biofilm-associated bacteria, offering an effective strategy for controlling biofilm-related infections by disrupting bacterial communication and adhesion., reducing virulence factor production, and degrade EPS components. In conclusion, the convergence of phytochemistry and nanotechnology provides a novel strategy for biofilm management. By overcoming existing limitations and focusing on clinical translation, phytochemical-based agents have the potential to significantly advance the treatment of biofilm-associated infections, contributing to the development of effective, sustainable, and safe antibiofilm therapies in the future.

Despite these encouraging developments, several challenges are existing for the clinical translation of phytochemical-based therapies. The challenges include the screening, extraction, and purification of active compounds, particularly those present in trace amounts. Moreover, real-world infections often involve polymicrobial biofilms and complex host interactions, and the current researches that predominantly focuses on monomicrobial biofilms cannot be generalized to clinical scenarios. Future research should focus on optimizing the extraction, purification, and standardization processes for natural phytochemicals to ensure reproducible efficacy. Advanced in vivo models mimicking clinical biofilm environments, including polymicrobial communities, are essential to better replicate clinical conditions and validate therapeutic potential. The integration of artificial intelligence and high-throughput screening technologies has the potential to expedite the identification of potent phytochemical candidates. Furthermore, the development of smart nanocarriers with capabilities for responsive drug release, specific targeting, and reduced toxicity will be critical for improving the safety and efficacy of phytochemicals.

## Data Availability

Data availability is not applicable to this article as no new data were created or analyzed in this study.

## Abbreviations

AIE: Aggregation-induced emission
AIP: Autoinducing peptide
BA: Baicalin
BBR: Berberine
BBR@SP gel: BBR encapsulated spirulina hydrogel
BBR-Rhe NPs: Berberine-Rhein nanoparticles
c-di-GMP: Cyclic dimeric guanosine monophosphate
CHR-BBR NPs: Chrysi-berberine nanoparticles
CDCS: β-Cyclodextrin grafted chitosan
CDCS-BA NPs: Baicalin-β-cyclodextrin grafted chitosan NPs
CNKI: China national knowledge infrastructure
Cur: Curcumin
Cur-NPs: Curcumin nanoparticles
eDNA: Extracellular DNA
EPS: Extracellular polymeric substances
Fmoc-F/BBR hydrogel: Fmoc-phenylalanine and berberine hybrid hydrogel
MSSA: Methicillin-sensitive *Staphylococcus aureus*
MRSA: Methicillin-resistant *Staphylococcus aureus*
MIC: Minimal inhibitory concentration
MBC: Minimum bactericidal concentration
MBIC: Minimal biofilm inhibitory concentration
MBEC: Minimal biofilm eradication concentration
NPs: Nanoparticles
OMP: Outer Membrane Protein
PIA: Polysaccharide intercellular adhesin
QS: Quorum sensing
ROS: Reactive oxygen species
SAR: Structure–activity relationships
SP: Spirulina
TA: Tanshinone IIA
TA@CS: Tanshinone IIA@Chitosan nanogels
UTIs: Urinary tract infections
